# Assessing the validity and intra-observer agreement of the MIDAM-LTC; an instrument measuring factors that influence personal dignity in long-term care facilities

**DOI:** 10.1186/1477-7525-12-17

**Published:** 2014-02-11

**Authors:** Mariska G Oosterveld-Vlug, H Roeline W Pasman, Isis E van Gennip, Henrica CW de Vet, Bregje D Onwuteaka-Philipsen

**Affiliations:** 1 Department of Public and Occupational Health, EMGO Institute for Health and Care Research, Expertise Center for Palliative Care, VU University Medical Center, Van der Boechorststraat 7, 1081 BT Amsterdam, the Netherlands; 2 Department of Epidemiology and Biostatistics, EMGO Institute for Health and Care Research, VU University Medical Center, Amsterdam, the Netherlands

**Keywords:** Dignity, Measurement instrument, Long-term care, Clinimetrics

## Abstract

**Background:**

Patients who are cared for in long-term care facilities are vulnerable to lose personal dignity. An instrument measuring factors that influence dignity can be used to better target dignity-conserving care to an individual patient, but no such instrument is yet available for the long-term care setting. The aim of this study was to create the Measurement Instrument for Dignity AMsterdam - for Long-Term Care facilities (MIDAM-LTC) and to assess its validity and intra-observer agreement.

**Methods:**

Thirteen items specific for the LTC setting were added to the earlier developed, more general MIDAM. The MIDAM-LTC consisted of 39 symptoms or experiences for which presence as well as influence on dignity were asked, and a single item score for overall personal dignity. Questionnaires containing the MIDAM-LTC were administered face-to-face at two moments (with a 1-week interval) to 95 nursing home residents residing on general medical wards of six nursing homes in the Netherlands. Constructs related to dignity (WHO Well-Being Five Index, quality of life and physical health status) were also measured. Ten residents answered the questions while thinking aloud. Content validity, construct validity and intra-observer agreement were examined.

**Results:**

Nine of the 39 items barely exerted influence on dignity. Eight of them could be omitted from the MIDAM-LTC, because the thinking aloud method revealed sensible explanations for their small influence on dignity. Residents reported that they missed no important items. Hypotheses to support construct validity, about the strength of correlations between on the one hand personal dignity and on the other hand well-being, quality of life or physical health status, were confirmed. On average, 83% of the scores given for each item’s influence on dignity were practically consistent over 1 week, and more than 80% of the residents gave consistent scores for the single item score for overall dignity.

**Conclusion:**

The MIDAM-LTC has good content validity, construct validity and intra-observer agreement. By omitting 8 items from the instrument, a good balance between comprehensiveness and feasibility is realised. The MIDAM-LTC allows researchers to examine the concept of dignity more closely in the LTC setting, and can assist caregivers in providing dignity-conserving care.

## Background

In light of the ageing population and the fact that people live a relatively longer period of time with chronic diseases and disabilities, concerns about losing personal dignity may increasingly arise [1,2].

*Personal* dignity is a type of dignity which relates to a sense of worthiness, is individualistic, tied to personal goals and social circumstances, and can be taken away or enhanced by circumstances or acts from others [3-5]. It should be distinguished from *basic* dignity, which is the inherent dignity of each human being and can be regarded as a universal and inalienable moral quality [1,6]. Earlier studies have shown that loss of personal dignity is associated with depression, hopelessness, a desire for death [7] and requests for euthanasia and physician-assisted suicide [8-11]. It is this type of dignity that is therefore important to understand, assess and preserve within the context of health care. The dignity concept can contribute to care in the last phase of life because it goes beyond assessment of physical and psychosocial health status and includes one’s perception of worthiness, both as an individual and in relation to close others and society [12-14]. By now, there is a substantial amount of knowledge on how patients nearing death [15-17] and older people in nursing homes [18-22] understand the concept of dignity. The majority of these studies had a qualitative design, and described the factors that can preserve or undermine personal dignity, and their interrelatedness.

Some of these empirical studies have served as a basis for the development of a measurement instrument for dignity. An example is the Patient Dignity Inventory, a 25-item list which was validated in patients in a palliative care program (predominantly cancer patients with a life expectancy of less than 6 months) [23,24]. Another instrument targeting at dying patients is the dignity card-sort tool, which can be used to rank factors influential in the loss or preservation of dignity at life’s end [25,26]. Recognizing the need for an instrument that is applicable to a more general patient population, our research group has developed the Measurement Instrument for Dignity AMsterdam (MIDAM) and examined its content validity in people with one or more advance directive(s) [27].

A setting for which no such measurement instrument is yet available is the long-term care setting. As compared to the general patient population, some aspects probably become more important for those who live permanently in an institution. Patients who are cared for in long-term care facilities not only face threats to dignity arising from functional and/or cognitive decline, they are also confronted with an unfamiliar living environment, little privacy, are often heavily reliant on staff and increasingly lack social networks, making them rather vulnerable to lose personal dignity [18,28]. A measurement instrument can give insight regarding those who are most at risk of losing dignity, and can be used to better target more effective, dignity-conserving care to an individual patient. Therefore, the aim of this study is to provide the long-term care setting with a valid and reliable measurement instrument. In this article we describe how the already existing MIDAM was adapted to create the MIDAM-LTC, and how we tested its content validity, construct validity and intra-observer agreement in a sample of 95 nursing home residents. Furthermore, we examined possibilities to reduce the length of the instrument, in order to make it feasible for use in practice.

## Methods

### Design and study population

The starting point for this study was our earlier developed measurement instrument for self-perceived dignity, retrospectively named MIDAM [27]. This instrument consists of 26 items (symptoms or experiences) categorized in 4 domains: (I) evaluation of self in relation to others, (II) functional status, (III) mental state and (IV) care and situational aspects. On the basis of the results of an extensive qualitative interview study among 30 nursing home residents [22,29], we added items specific for long-term care facilities to the MIDAM, i.e. items with regard to the way residents are treated by nursing home staff, living circumstances in the nursing home, living in a group, limited capacity of nursing home staff, sense of belonging and sense of meaning (see Table 1). These six themes were frequently mentioned by nursing home residents in the interview study, but not adequately reflected in the MIDAM. In a process of reflection and interaction, we formulated 13 items following the structure of the original MIDAM-items. We abundantly added items in order to be comprehensive. The extension “for Long-Term Care facilities” was added to the name of the instrument; MIDAM-LTC (see Additional file 1).

**Table 1:** Origin of items added to the MIDAM in order to create the MIDAM-LTC

**Theme from qualitative study[**[22]**,**[29]**]**	**Items already in MIDAM[**[27]**]**	**Items added to create MIDAM-LTC**
Treatment of nursing home staff	- I am not treated with enough respect by caregivers (e.g. doctors, nurses)	- I feel I’m not being taken seriously because of my age or illness
	- Doctors do not pay enough attention to my wishes	- The nurses have little time for me
Living circumstances in nursing home	- I have insufficient opportunity to live as I wish according to my beliefs or religion	- I miss my home, my things, my loved ones and the familiar environment that I left behind
		- I have little opportunity to shower
		- My room in the nursing home is small
		- I find it difficult to adjust to the rhythm and habits of the nursing home
Living in a group	- I have little privacy	- I find it difficult to eat or sit in the living room with 'unknown’ others
		- I have little contact with the other nursing home residents
Limited capacity of nursing home staff		- I often have to wait a long time before I receive help
		- There are a lot of different nurses caring for me
		- I feel guilty if I have to call on the nurses a lot
Sense of belonging to others	- I receive little attention and care from the people around me	- I no longer feel part of society
	- I feel worthless for my friends and family	
Sense of meaning	- I have the feeling that I have not made any meaning or lasting contribution during my lifetime	- I am bored and every day feels the same to me

To test the instrument’s psychometric properties, data were collected on the general medical wards (long-stay units for people with physical illnesses) of six nursing homes in the Netherlands. Nursing home residents were recruited with help from a unit manager, elderly care physician or the most important nurse on the ward. Eligible residents were all those on the wards who were cognitively able to understand the instructions and questions. Because many nursing home residents could not write anymore, an additional criterion was that a resident had to be able to communicate with an interviewer who would administer the questionnaire face-to-face and fill in the answers. All eligible residents received an information letter approximately one week before the interviewers came to the nursing home, and they could indicate whether or not they wanted to participate in the study at the time the interviewer visited them one week later. By consenting to participate in the study, the nursing home resident also gave us permission to ask his/her contact person in the care record (closest relative), elderly care physician and responsible nurse for information about the mental and physical health status of the resident.

### The questionnaire

For each item (symptom or experience) in the MIDAM-LTC, respondents were first asked whether it applied to their life (by thinking about the past 2 days). Only if they answered affirmatively, they were asked to what extent this influenced their sense of dignity on a five-point scale (see Table 2). We deliberately distinguished between these two questions, in order to prevent respondents not suffering from a certain symptom or experience rating its putative association with dignity. In addition, we asked respondents to rate their sense of dignity on a 10-point scale (1 = sense of dignity completely lost, 10 = sense of dignity completely intact). Besides these questions about personal dignity, the questionnaire asked for respondent characteristics, and contained measurements of related constructs like the WHO-Five Well-being Index, the EQ-5D, and a question to rate quality of life (on a 10-point scale). In order to assess the resident’s mental and physical health status, the Cognitive Performance Scale [30], Barthel Index [31] and Karnofsky Performance Status Scale [32] were presented to the nurses. The elderly care physician and closest relative provided information about the resident’s diseases.

**Table 2:** Questions for each item in the MIDAM-LTC


*For each item the following two questions can be asked:*							
a. Does this apply to you? (think about the past 2 days)							
	No		Yes				
b. If so, to what extent does this influence your sense of dignity?							
Not at all		A little	Somewhat		Quite a lot		Very much
1		2	3		4		5
*Example:*							
	**a. Does this apply to you?**		**b. Does this influence your sense of dignity?**				
			Not at all ← →Very much				
**I am incontinent**	❑ No ↓ (item 2)	❑ Yes → (question b)	1	2	3	4	5
**I have little privacy**	❑ No ↓	❑ Yes →	1	2	3	4	5

### Procedure

We first piloted the questionnaire with three nursing home residents, after which a few items of the MIDAM-LTC were reformulated. Subsequently, the questionnaire was administered face-to-face to all participants by four interviewers (among whom MOV and IG) and took approximately 30 minutes for all questions, of which the MIDAM-LTC lasted about 20 minutes. Residents were handed a card with the answering options to help them choosing their answer. Ten residents were asked to fill in the questionnaire by using the 'think aloud’ method [33]. This method was used to elicit data on nursing home residents’ thought processes as they responded to the items and to assess whether they understood the questions as we intended them. To be able to study intra-observer agreement, we visited the nursing homes one week later and asked 49 residents to fill in the questionnaire a second time with help from another interviewer than the week before. We intended to have two measurements of half of the participants, and asked the residents who were available at the time of our second visit. Only a few declined to participate twice. We chose for a time interval of one week, which we estimated to be long enough to prevent recall, though short enough to prevent that major changes in personal dignity occurred [34]. The study was approved by the Medical Ethics Committee of the VU University Medical Center.

### Analysis of psychometric properties

Each item of the MIDAM-LTC can be considered a causal indicator, meaning that items are not expected to correlate with each other, and that even a single symptom or experience may suffice to undermine dignity, while a low sense of dignity need not necessarily imply that someone suffers from all the symptoms listed [35,36]. Therefore, performing a factor analysis or calculating a total score of all items was not meaningful. Instead, each item had to be considered separately, while we kept in mind that the instrument needed to be comprehensive, though take as little effort and time as possible to be filled in, in order to be feasible for use in practice.

Content validity was determined through two approaches. To examine whether all items in the MIDAM-LTC were relevant for personal dignity, we first calculated the mean scores per item for influence on dignity and the percentage of respondents who indicated that the item (if present) influenced their dignity quite a lot or very much (score 4 or 5). Because a valid ground for omitting items in models with causal indicators is that they occur too infrequently to be worth reporting [36], we decided to omit those items which fulfilled two criteria: a mean score for influence on dignity lower than 2.50, and a percentage below 25% of respondents who indicated that the item influenced their dignity quite a lot or very much (score 4 or 5). Comprehensiveness was assessed by asking the residents who answered the questionnaire while thinking aloud whether they missed any items that influenced their dignity.

To assess construct validity, we tested several hypotheses about the relation between dignity and related constructs (Table 3), based on expectations arising from our qualitative interview study. Pearson’s correlation coefficients between the constructs were calculated. According to Cohen, we classified a correlation coefficient over 0.5 as a strong relation, 0.3 to 0.5 as moderate, 0.1 to 0.3 as small, and below 0.1 as no relation between constructs at all [37].

**Table 3:** Hypotheses to assess construct validity

**Hypothesis**	**Explanation**
1. The number of items where people indicate that it influences their dignity (very) much (score 4 or 5) correlates strongly with the single item score for overall personal dignity (on a scale from 1 to 10).	Although even a single symptom or experience may suffice to violate dignity for an individual nursing home resident, we expect that – on study population level – the more items influence dignity to a large extent, the lower the single item score for personal dignity.
2. Both the score for quality of life (on a scale from 1 to 10) and the score on the WHO Well-Being Scale (on a scale from 1 to 100) correlate moderately to strong with the single item score for personal dignity (on a scale from 1 to 10), though the correlation with the WHO Well-Being Scale is stronger than with the score for quality of life.	These expectations arise from the results of our interview study [22,29], in which we noticed that many nursing home residents associated 'quality of life’ with their physical health status. Because personal dignity encompasses relational aspects as well, we expect 'well-being’ – which might have a more holistic connotation – to be more closely related to the concept of dignity.
3. Both the score on the Karnofsky Performance Status Scale and the score on the Barthel Index correlate low to moderately with the single item score for personal dignity (on a scale from 1 to 10), though the correlation with the Barthel Index is stronger than with the Karnofsky Performance Status Scale.	Whereas the Barthel Index measures physical functioning on 10 Activities of Daily Living – and the Karnofsky Performance Status Scale simply by one question – we expect more variation in the scores on the Barthel Index, and therefore a stronger correlation with personal dignity. However, we expect these low to moderate correlations since physical functioning is only one aspect of personal dignity.

In examining the intra-observer agreement after one week, we were especially interested in absolute measures of agreement. Since Intraclass Correlation Coefficients and Cohen’s kappa values are relative measures of agreement [38], we calculated agreement percentages between the two observations. First, we compared the single item scores for overall personal dignity on both measurements. Next, percentages of agreement were calculated for each item’s influence on dignity in two different ways; we distinguished between exact agreement and agreement if we allowed the scores to differ one point on the 5-point scale. We hereby reasoned that an item could not undermine dignity if not present in a resident’s life - a non-affirmative answer on the first question - and scored it accordingly on the second question (score 1 on the 5-point scale). In this way, we could also take the first question regarding presence into account in calculating the agreement percentages. In addition, we looked at each item’s mean score for influence on dignity on both measurements, and tested whether these differed from each other with paired sample t-tests. Finally, average scores and average agreement percentages across all items’ influence on dignity were calculated.

## Results

### Sample characteristics

In total, 131 residents were approached to participate in this study. Twenty-one residents declined to participate, two residents were absent on the days the interviewers were in the nursing home and two residents died after they had received the information letter. A further eleven residents were excluded by the time of the interview, as it turned out that they were cognitively unable to understand the questions. This resulted in 95 participating residents, whose characteristics are shown in Table 4. The majority of the respondents were female and they averagely resided 744 days (median 575 days) in the nursing home. The most frequently reported diseases were heart diseases, rheumatoid arthritis and stroke. Respondents rated their sense of dignity on average as 7.3 (SD 1.6) on the 10-point scale.

**Table 4:** Characteristics of the study population

**Characteristics**	**N = 95**^**1**^
Sex, female (%)	65
Age, mean (SD) [range]	79.8 (11.2) [44 – 100]
Cultural background (%)	
Dutch	88
Surinam	8
Other	4
Diseases (%)^2^	
Heart diseases	36
Rheumatoid arthritis	29
Stroke	28
Diabetes	25
Cancer	14
Asthma/COPD	11
Depression	8
Parkinson’s disease	5
Multiple Sclerosis	3
Length of stay in nursing home, mean days (SD) [range]	744 (764) [29 – 3830]
Cognitive Performance Scale (%)	
Intact	73
Borderline intact	21
Mild impairment	3
Moderate impairment	2
Moderate severe impairment	1
Having a belief/religion that is appreciated as important (%)	45
Barthel Index, mean (SD) [range]^3^	7.4 (4.9) [0 – 19]
WHO-Five Well Being Index, mean (SD) [range]^4^	54.8 (23.5) [0 – 100]
EQ-5D, mean (SD) [range]^5^	0.33 (0.26) [-0.2 – 1]
Quality of life, mean (SD) [range]	6.6 (2.0) [1 – 10]
Single item score for personal dignity, mean (SD) [range]	7.3 (1.6) [1 – 10]

^1^Missing observations for 2–10 respondents: WHO-Five Well Being Scale (2), EQ-5D (2), Quality of life (3), Single item for personal dignity (3), Cognitive Performance Scale (3), Barthel Index (4), Diseases (8), Length of stay (10).

^2^Many respondents had several diseases. The most prevalent diseases are listed in the table, but many others were mentioned (e.g. Chron’s disease, cataract, pneumonia, polyneuropathy, ALS, aneurysma hydrocephalus, epilepsy).

^3^The Barthel Index assesses ability to perform activities of daily living: 0 = total dependence – 20 = maximum independence.

^4^A higher score on the WHO-Five Well Being Index (from 0 to 100) indicates more well-being.

^5^The EQ-5D assesses health-related quality of life on 5 dimensions 'mobility, self-care, usual activities, pain/discomfort and anxiety/depression’: -0.33 = severely disabled on all domains – 1 = perfect health.

### Content validity

In Table 5, the items are ranked according to the mean scores given for influence on dignity, and ordered per domain. The percentage of nursing home residents who agreed that an item applied to their life ranged from 98.9% ('Using medical-technical aids’) to 5.3% ('Not looking well-groomed’ and 'Not made any meaning or lasting contribution’) and were highest in the domain 'Functional status’. Most of the 13 added items specific for long-term care facilities were frequently present in the study population. However, their mean influence on sense of dignity was generally rather small. The highest mean scores for influence on dignity could instead be found in the domain 'Evaluation of self in relation to others’, and ranged from 3.25 ('Feeling worthless for friends and family’ and 'Not treated with respect by caregivers’) to 1.87 ('A changed physical appearance’). Nine items barely exerted influence on dignity according to the formulated criteria (marked with an asterisk in Table 5). Five of them were items that were specifically added for the long-term care setting.

**Table 5:** Presence and influence on dignity per item in a group of nursing home residents (N = 95), ordered per domain

**Item**	**Yes, present**^**1**^**%**	**Influence on dignity**^**2**^	
		**Mean**	**% (very) much**
(I) Evaluation of self in relation to others			
Feeling worthless for friends and family	16.8	3.25	43.8
Not looking well-groomed	5.3	3.20	40.0
Very little self-respect	16.8	3.00	35.7
Not made any meaning or lasting contribution	5.3	3.00	40.0
Lost control over my life	43.2	2.78	34.1
Not able to oversee what’s happening to me	32.6	2.77	35.5
No longer feeling like the person I was before	55.8	2.58	30.2
A changed physical appearance*	25.3	1.87	16.7
(II) Functional status			
Not able to carry out usual activities	48.9	3.17	58.7
Impaired sight despite using aids	29.5	3.07	57.1
Incontinence	53.7	2.80	35.3
Not able to wash, dress or go to the toilet independently	83.2	2.59	24.1
Not able to do domestic tasks	95.8	2.58	27.5
Hampered communication due to impaired hearing/speech	30.5	2.55	34.5
Physical complaints	67.4	2.55	29.7
Using medical-technical aids	98.9	2.38	29.8
Not able to eat and drink independently*	7.4	2.14	14.3
(III) Mental state			
Lost fighting spirit	26.3	3.00	44.0
Feeling depressed	31.6	2.87	26.7
Mentally unable to take decisions**	9.5	2.44	22.2
Being forgetful*	47.4	2.24	20.0
(IV) Care and situational aspects			
Not treated with respect by caregivers	16.8	3.25	50.0
Little privacy	37.9	2.94	38.9
Receiving little attention for my wishes from doctors	23.7	2.86	36.4
Insufficient opportunity to live according to beliefs or religion	6.3	2.83	33.3
Receiving little attention and care from people around me	17.9	2.38	25.0
Added items specific for LTC setting			
Not being taken seriously	21.3	3.10	40.0
Difficulties with adjusting to the nursing home	23.2	2.95	45.5
Feeling bored and experiencing every day as the same	36.2	2.74	38.2
Missing the things I left behind	80.9	2.62	36.8
Feeling guilty about calling on the nurses a lot	57.4	2.56	24.1
Waiting a long time for help	58.9	2.53	29.1
No longer feeling part of society	55.3	2.50	32.7
Receiving little time from nurses*	55.8	2.43	22.6
Little opportunity to shower*	38.9	2.33	22.2
Difficult to be in living room with 'unknown’ others	22.3	2.25	25.0
A lot of different nurses*	61.7	2.24	19.0
Small room in nursing home*	37.6	2.09	20.6
Having little contact with other residents*	58.5	2.02	20.4

^1^Presence: between 0 and 2 missing observations per item.

^2^Influence on dignity: between 0 and 2 missing observations per item (of the respondents who indicated that an item applied to their life).

*For this item the mean score for influence on dignity is lower than 2.50, and less than 25% of the nursing home residents to whom the item applied indicated this to influence their dignity quite a lot or very much (score 4 or 5); this item will therefore be removed from the instrument.

**Although the criteria for omission are met by this item, we reasoned to keep this item in the instrument in a reformulated phrase.

Before definitively removing these nine items from the instrument, we listened to the recordings of the 10 nursing home residents who filled in the MIDAM-LTC while thinking aloud, to find out the reason for the small extent to which an item averagely influenced dignity. This analysis revealed that some symptoms or experiences, although present in a resident’s life, did not undermine dignity because the resident was satisfied with the way it was. For example, the majority of the residents who agreed that they had a small room did not long for a bigger room *(“Where do I need it for?”).* More or less the same argumentation was given for the small influence on dignity for the items 'Little opportunity to shower’ *(“I don’t want to shower more often, it makes me tired”)* and 'Having little contact with other residents’ *(“They are too demented or fighting with each other, so I’d rather be on my own”)*. The items 'Receiving little time from nurses’ and 'A lot of different nurses’ hardly undermined dignity, because residents ascribed the presence of both items to the circumstances rather than to nurses’ unwillingness *(“They are terribly busy because of the lack of staff. If I really need them, they will make time for me”)*. 'Being forgetful’ and 'A changed appearance’ were regarded and accepted as belonging to the ageing process and therefore not undermining dignity *(“Yes I have more wrinkles, grey hair and I forget things occasionally, but may I? I’m 82 years old!”).* An extra reason why 'A changed appearance’ barely exerted influence on dignity was that some nursing home residents said they had lost weight, which they actually regarded as positive. There was one item for which no sensible explanation could be found for the low scores on a) presence and b) influence on dignity: 'Mentally unable to take decisions’. Possibly, these low scores were a consequence of our decision to include only respondents who were cognitively able to understand the questions. It might also be that the way the item was phrased could have discouraged respondents to indicate the item applied to them. We therefore chose to keep this item in the instrument and reformulate it more mildly into 'I feel unable to take major decisions’.

In addition, the thinking aloud method revealed that the item 'Feeling bored and experiencing every day as the same’ consisted of two different aspects; nursing home residents could experience every day as the same, while not feeling bored. Therefore, we decided to reformulate this item into 'All days seem colourless to me’. Furthermore, the items 'Not able to carry out usual activities’ and 'Lost fighting spirit’ were differently interpreted by the residents. For some, usual activities were activities they used to do in the past (e.g. cycling), whereas others thought of activities they did in the nursing home (e.g. reading and watching TV). To correct for these different interpretations, and to only include those activities participants have a current need for, this items needs to be reformulated into 'Not able to carry out activities I would like to do’. As for fighting spirit, some interpreted this as 'enjoying all organized activities in the nursing home’, whereas others considered this as 'standing up for themselves’. However, this latter item does not need to be reworded, as it concerns the same character trait that lies at the root of these two different manifestations. Since the purpose of the MIDAM-LTC is to give insight regarding those who are most at risk of losing dignity, high scores on items are merely a signal to start questioning the source of dignity-related distress. Finally, no items were missed by the nursing home residents.

### Construct validity

We tested our first hypothesis (see Table 3) without the eight items of which we had decided to omit them, as described above. The number of items where residents indicated that it influenced their dignity (very) much (score 4 or 5) correlated moderately with the single item score for overall personal dignity (r = -0.49), just missing the threshold to be classified as a strong correlation. Our second hypothesis was supported by the data: Pearson’s r for the relation between the single item scores for quality of life and personal dignity was 0.50, and between the WHO Well-Being Scale and personal dignity 0.53. Unfortunately, we could test our third hypothesis only partially. According to our expectations, the correlation between the Barthel Index and personal dignity was low (r = 0.23). However, scores on the Karnofsky Performance Status Scale were sometimes unrealistically high (for some residents even '100’ which means perfectly healthy), indicating that some nurses did probably not understand the question or took the average nursing home residents as a reference in mind when answering this question. Calculating a correlation coefficient between this scale and the single item score for dignity was therefore not appropriate.

### Intra-observer agreement

Table 6 shows the percentages of agreement for each item’s influence on dignity, as well as average agreement percentages across all items of the MIDAM-LTC (without the eight items of which we had decided to omit them). The average exact agreement percentage for all items combined was 70.6% and increased to 83.4% when we allowed one point difference on the five-point scale. In the latter condition, individual item’s agreement ranged from 59.6% to 95.8%. No significant differences between the mean scores for influence on dignity on both measurements existed; neither for all items combined, nor for any individual item (data not shown). Of all nursing home residents who rated their overall personal dignity on both measurements, 50.0% gave the exact same score on the single item 10-point scale. A further 30.4% of the residents differed only one point in their ratings.

**Table 6:** Intra-observer agreement after 1 week in a group of nursing home residents (N = 49)

**Item**^**1**^	**Influence on dignity**^**2**^	
	**% exact agreement**	**% agreement if 1 point difference allowed**^**3**^
(I) Evaluation of self in relation to others		
Feeling worthless for friends and family	85.1	95.7
Not looking well-groomed	93.8	95.8
Very little self-respect	75.0	89.6
Not made any meaning or lasting contribution	87.8	87.8
Lost control over my life	72.9	85.4
Not able to oversee what’s happening to me	77.1	85.4
No longer feeling like the person I was before	56.3	75.0
(II) Functional status		
Not able to carry out usual activities	58.3	72.9
Impaired sight despite using aids	85.7	93.9
Incontinence	61.2	85.7
Not able to wash, dress or go to the toilet independently	59.2	81.6
Not able to do domestic tasks	38.8	65.3
Hampered communication due to impaired hearing/speech	85.7	93.9
Physical complaints	47.9	70.8
Using medical-technical aids	28.6	71.4
(III) Mental state		
Lost fighting spirit	74.5	80.9
Feeling depressed	75.0	89.6
Mentally unable to take decisions	93.8	95.8
(IV) Care and situational aspects		
Not treated with respect by caregivers	79.2	91.7
Little privacy	64.6	77.1
Receiving little attention for my wishes from doctors	80.0	86.7
Insufficient opportunity to live according to beliefs or religion	89.4	91.5
Receiving little attention and care from people around me	87.2	89.4
Added items specific for LTC setting		
Not being taken seriously	76.6	83.0
Difficulties with adjusting to the nursing home	83.0	89.4
Feeling bored and experiencing every day as the same	65.2	80.4
Missing the things I left behind	42.6	59.6
Feeling guilty about calling on the nurses a lot	50.0	73.9
Waiting a long time for help	60.9	84.8
No longer feeling part of society	76.6	85.1
Difficult to be in living room with 'unknown’ others	78.7	83.0
Average % agreement (across all 31 items)	70.6	83.4

^1^Between 0 and 4 missing observations per item.

^2^Before calculating these percentages, items were recoded to exert no influence on dignity (score 1 on the 5-point scale) if they did not apply to a nursing home resident.

^3^'Agreement’ is expanded here and also includes those cases where the extent to which an item influenced a resident’s dignity differed only 1 point (on the 5-point scale) between both measurements.

## Discussion

By implementing all changes and omitting some items, the revised MIDAM-LTC consists of 31 items (see Additional file 1), and a good balance between comprehensiveness and feasibility is realised. The MIDAM-LTC has good basic standard psychometric properties. Content validity refers to the extent to which the concept of interest is comprehensively represented by the items of an instrument. That no aspects were missed provides evidence for a good content validity of the MIDAM-LTC. In addition, the 13 added items specific for long-term care facilities were derived from an extensive qualitative interview study with 30 nursing home residents [22,29], so these items were considered relevant by the target population. Given that we abundantly added items in our efforts to be comprehensive, it is therefore not surprising that five of these 13 added items were not found to have a large influence on personal dignity (although frequently present) and could be omitted from the instrument. In contrast, only three items from the general MIDAM [27] were found to barely influence dignity, demonstrating the validity of these already existing items across different settings.

Construct validity, applicable in situations in which there is no gold standard, refers to whether the instrument provides the expected scores, based on existing knowledge about the construct [39]. Our expectations regarding the extent to which personal dignity correlated with other constructs were virtually all supported by the data, and the one expectation that was not (formulated in the first hypothesis) came very close to confirmation. This shows that the MIDAM-LTC has good construct validity. Although dignity appears strongly related to quality of life, it is noteworthy that nursing home residents rated their dignity generally higher (with a mean of 7.3 out of 10) as compared to their quality of life (with a mean of 6.6 out of 10). Firstly, this finding suggests that personal dignity is a resilient construct. Most nursing home residents seem able to withstand the various physical and psychological challenges they face, making a great undermining of dignity rather the exception than the norm. Secondly, this higher score for dignity suggests that nursing home residents can distinguish between personal dignity and quality of life, despite the overlap in physical, socio-psychological and spiritual aspects reflected in both. Whereas overall quality of life exceeds health-related quality of life, which in turn is more than health status only [40], dignity may go beyond quality of life because it also brings one’s perception of being worth of respect from themselves and from others along. As quality of life is defined as a subjective integration of all aspects of one’s life deemed relevant [41], personal dignity may stress more importance on the evaluation of oneself in close relation to others. In our qualitative studies, we found that relational and societal aspects could undermine a resident’s dignity, but preserve or enhance it as well [22,29]. For example, being socially involved with others, receiving good professional care and social support, and being amongst disabled others and therefore less prone to exposures of disrespect from the outer world could help residents to maintain or regain their dignity. Presence of these preservative factors may explain the relatively high score given for personal dignity in this study.

An adequate intra-observer agreement is attributable to more than 83% of the residents who gave a practically consistent score for the each item’s influence on dignity over a week, and to more than 80% of the residents who did this for the single item score for overall personal dignity. Some lower agreement scores were found for individual items that were more prone to fluctuate in time (e.g. 'feeling guilty about calling on the nurses a lot’). Similar good results were obtained in a study on the psychometric properties of the Patient Dignity Inventory [24]. Test-retest reliability was then measured over a 24-hour time frame, which might be too short to ensure no recall bias was introduced. That we found these high percentages even after one week implies that personal dignity and the items in the MIDAM-LTC are quite stable.

### Strengths and limitations

The MIDAM-LTC is, to our knowledge, the first instrument measuring dignity specifically targeted at the population living in long-term care facilities. Although nursing homes are only one facility providing long-term care, we believe that the MIDAM-LTC is relevant for all people living in any kind of long-term care institution. Since the adjustment of the instrument is based upon the perspectives of nursing home residents - who are probably the most severely disabled patients within the long-term care setting - it is likely that the added items cover the whole range of relevant aspects influential to the dignity of persons living in institutions. By using the think-aloud method in 10 nursing home residents, we gained valuable insight in the thought processes they engaged in when responding to the questionnaire. However, in interpreting their answers, we must be aware that answers obtained by the think-aloud method may be more social-desirable than the answers received from the other nursing home residents. Our study was limited to the experiences of residents who were able to think and communicate about dignity. Although applicable to people who suffer from mild cognitive decline, the instrument cannot provide relevant information in people with more severe cognitive incapacities. Another limitation is that the MIDAM-LTC can only detect whether a certain symptom or experience undermines personal dignity, and not what preserves dignity. It might be that the absence of certain symptoms or experiences could actually have improved one’s sense of dignity, e.g. if a resident is one of the few still being able to go to the toilet independently. However, an instrument measuring both undermining and preservative factors would require a different structure, and would possibly become too complex to be understood by respondents. To provide the long-term care setting with a feasible instrument, we therefore only focused on the factors that undermine dignity, as they are more relevant for practice.

## Conclusions

The MIDAM-LTC appears to be a reliable and valid instrument for the assessment of factors influencing personal dignity in the long-term care setting. By reducing the number of items listed, the feasibility of the instrument for use in practice has increased. It allows researchers to examine the concept of dignity more closely in long-term care, e.g. by investigating distributions of sources of dignity-related distress across various patients. Caregivers working in long-term care institutions could use the MIDAM-LTC to assist them in providing dignity-conserving care, by identifying the factors that undermine a patient’s personal dignity.

## Competing interests

The authors declare that they have no competing interests.

## Authors’ contributions

BOP conceived of the study, and all authors participated in its design. MOV and IG collected the data. MOV performed the analyses and drafted the manuscript. RP, HdV, IG and BOP regularly met with MOV to discuss and interpret the findings. All authors read and approved the final manuscript.

## Supplementary Material

Additional file 1 The MIDAM-LTC. Click here for file
