# Supplementary material for: Assessing the validity and intra-observer agreement of the MIDAM-LTC; an instrument measuring factors that influence personal dignity in long-term care facilities
Source: Health Qual Life Outcomes. 2014 Feb 11;12:17. doi: 10.1186/1477-7525-12-17 (PMC3930004; doi:10.1186/1477-7525-12-17)
Supplement: Additional file 1 — The MIDAM-LTC. [file 1477-7525-12-17-S1.doc]

**Additional file 1**

**The MIDAM-LTC**

We would like to present you a relatively long list of items (situations, complaints, feelings and thoughts). About each item, we ask you two questions:

a) Does this apply to you? (think about the past 2 days)

b) If so, to what extent does this influence your sense of dignity?

If an item does not apply to you, please continue to the next item on the list. However, if an item does apply to you we would like you to answer question b.

| For each question b you can choose from 5 answers:  1 = Not at all 2 = A little 3 = Somewhat 4 = Quite a lot 5 = Very much |
| --- |

**General list (MIDAM)**

*Evaluation of self in relation to others*

- I have the feeling that I can’t quite oversee what is happening to me
- I no longer feel like the person I was before
- I have the feeling that I have not made any meaning or lasting contribution during my lifetime
- I have lost control over my life
- I feel worthless for my friends and family
- I have very little self-respect
- I don’t look well-groomed

*Functional status*

- I am not really able to wash, dress or go to the toilet independently
- I am incontinent (have difficulty in containing my urine/faeces)
- I am not really able to do domestic tasks by myself (e.g. cooking, cleaning, shopping)
- I have physical complaints (e.g. pain, shortness of breath, nausea, constipation, itch)
- In daily life I have to use medical-technical aids (e.g. wheelchair, stoma, oxygen cylinder)
- I am not really able to carry out the activities that I would like to (instead of: I am not really able to carry out my usual activities (e.g. read the newspaper, hobbies, job)
- Even wearing glasses or lenses I am not able to see very well
- My impaired hearing or speech hampers me in the communication with others

*Mental state*

- I feel unable to make major decisions (instead of: I am mentally not really able to take decisions on my own)
- I feel depressed
- I have lost my fighting spirit

*Care and situational aspects*

- I have insufficient opportunity to live as I wish according to my beliefs or religion
- I have little privacy
- I receive little attention and care from the people around me
- I am not treated with enough respect by caregivers (e.g. doctors, nurses)
- Doctors do not pay enough attention to my wishes

**Items added – specific for Long-Term Care**

- I find it difficult to adjust to the rhythm and habits of the nursing home
- I often have to wait a long time before I receive help
- I feel guilty if I have to call on the nurses a lot
- I miss my home, my things, my loved ones and the familiar environment that I left behind
- I feel I’m not being taken seriously because of my age or illness
- I no longer feel part of society
- All days seem colourless to me (instead of: I am bored and every day feels the same to me)
- I find it difficult to eat or sit in the living room with ‘unknown’ others

**Items eliminated from the MIDAM-LTC because they barely exerted influence on dignity**

- I am not really able to eat and drink independently
- My physical appearance has changed (e.g. hair loss, severe loss of weight)
- I am forgetful
- The nurses have little time for me
- I have little opportunity to shower
- There are a lot of different nurses caring for me
- My room in the nursing home is small
- I have little contact with the other nursing home residents

**General question**

Thinking about the past 2 days, can you describe your sense of dignity? (please put a circle around the appropriate number on the scale)

| Sense of dignity  completely lost | 1 - 2 - 3 - 4 - 5 - 6 - 7 - 8 - 9 - 10 | Sense of dignity  completely intact |
| --- | --- | --- |
